# Central Thalamic Deep Brain Stimulation Modulates Autonomic Nervous System Responsiveness in Disorders of Consciousness

**DOI:** 10.1111/cns.70274

**Published:** 2025-03-06

**Authors:** Tianqing Cao, Xiaoke Chai, Hongbin Wu, Nan Wang, Jiuxiang Song, Qiheng He, Sipeng Zhu, Yitong Jia, Yi Yang, Jizong Zhao

**Affiliations:** ^1^ Department of Neurosurgery, Beijing Tiantan Hospital Capital Medical University Beijing China; ^2^ China National Clinical Research Center for Neurological Diseases Beijing China; ^3^ School of Advanced Manufacturing Nanchang University Nanchang Jiangxi China; ^4^ Chinese Institute for Brain Research Beijing China; ^5^ Beijing Institute of Brain Disorders Beijing China

**Keywords:** autonomic nervous system, central autonomic network, deep brain stimulation, disorders of consciousness, heart rate variability

## Abstract

**Background:**

The heart rate variability (HRV) of patients with disorders of consciousness (DOC) differs from healthy individuals. However, there is rarely research on HRV among DOC patients following treatment with deep brain stimulation (DBS). This study aims to investigate the modulatory effects of DBS‐on the central‐autonomic nervous system of DOC based on the study of HRV variations.

**Methods:**

We conducted DBS surgery on eight patients with DOC. Postoperatively, all patients underwent short‐duration stimulation for 3 days, with stimulation frequencies of 25 Hz, 50 Hz, and 100 Hz respectively. Each day comprised four cycles, with a stimulation duration of 30 min DBS‐on and 90 min DBS‐off. We obtained the coma recovery scale‐revised (CRS‐R) scores and synchronously recorded electrocardiographic data.

**Resulits:**

We analyzed the HRV indices, including time‐domain and frequency‐domain parameters across various time points for all patients. The HRV exhibited a consistent trend across the three groups with different parameters. Notably, the most pronounced HRV changes were induced by the 100 Hz. Long‐term follow‐up indicates that high‐frequency (HF), low‐frequency (LF), and total power (TP) of HRV may serve as predictive indicators in the prognosis of patients.

**Conclusion:**

Our study reveals that DBS enhances DOC patient consciousness while increasing HRV. Specifically, frequency‐domain indices correlate with favorable prognosis.

## Graphical abstract

Central thalamus deep brain stimulation (DBS) exerts a regulatory effect on the autonomic nervous system in patients with disorders of consciousness (DOC). We analyzed the HRV indices, including time‐domain and frequency‐domain parameters across various time points for all patients. The HRV exhibited a consistent trend across the three groups with different parameters. Notably, the most pronounced HRV changes were induced by the 100 Hz. Long‐term follow‐up indicates that high‐frequency (HF), low‐frequency (LF), and total power (TP) of HRV may serve as predictive indicators in the prognosis of patients.

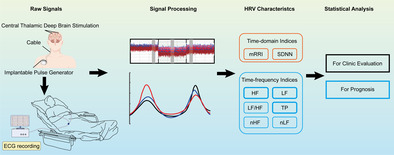

## 1 Introduction

Disorders of consciousness (DOC) refer to a state of impaired consciousness function lasting for more than 28 days, typically arising from traumatic brain injury (TBI), cerebral hemorrhage, or hypoxic–ischemic encephalopathy [1]. Based on the degree of impairment in arousal and awareness, DOC can be categorized into three types: coma, unresponsive wakefulness syndrome/vegetative state (UWS/VS, where patients are awake but unable to demonstrate any conscious responses) [2, 3, 4], and minimally conscious state (MCS, where patients exhibit minimal but discernible signs of consciousness) [5]. MCS was subcategorized based on the complexity of patients' behaviors: MCS+ describes high‐level behavioral responses (i.e., command following, intelligible verbalizations or non‐functional communication) and MCS− describes low‐level behavioral responses (i.e., visual pursuit, localization of noxious stimulation or contingent behavior such as appropriate smiling or crying to emotional stimuli) [6]. After severe impairment of consciousness, the possibility of spontaneous recovery for patients appears to be exceedingly slim [7]. Currently, there is a lack of effective and universally accepted strategies to promote awareness and arousal. This current situation poses significant challenges for the management of DOC patients, not only for their families but also for the entire healthcare system. DBS emerges as a promising approach, having already demonstrated efficacy in the treatment of Parkinson's disease and epilepsy [8, 9]. DBS of the central thalamus holds great promise as one of the neuromodulation methods for treating DOC. Schiff et al. reported in 2007 the positive behavioral outcomes observed in a patient with TBI who underwent DBS targeting the central thalamus (CT‐DBS) [10]. In a recent Phase I clinical trial, DBS surgery was performed on the centromedian‐lateral nucleus and associated dorsal tegmental tract of medial thalamus (CL/DTTm) to enhance prefrontal/frontal cortical and striatal inputs in patients with moderate to severe traumatic brain injury (msTBI), with the aim of improving executive control abilities [11].

The autonomic nervous system (ANS) meticulously monitors and regulates a vast and intricate network of distributed biological sensors, controlling arterial blood pressure and local blood flow in various organs of the human body in response to tissue metabolic demands, thereby maintaining life stability [12, 13, 14, 15]. The cardiovascular center receives integrated inputs from the telencephalon, diencephalon, and brainstem, and orchestrates the generation of stimulus‐specific autonomic response patterns through descending projections of sympathetic and parasympathetic neurons at the spinal cord level [16, 17]. Concurrently, the cardiovascular center conveys peripheral mechanical stimuli and hormonal regulatory signals upwards via the nucleus of the solitary tract (NTS), projecting them to the interconnected limbic cortical regions and the medial prefrontal cortex within subcortical nuclei, forming a feedback loop that collectively regulates the cardiovascular system. This regulatory pattern is known as the central autonomic network (CAN) model [12, 18, 19]. The thalamus, as a pivotal hub for information processing and transmission, influences autonomic nervous system activity by processing inputs from the NTS and other sensory nuclei within the medulla oblongata [20, 21]. Yamamoto and Tsubokawa et al. were observed that the activation of DBS targeting the central thalamus elicited a robust arousal response, accompanied by significant increases in regional r‐CBF (regional cerebral blood flow), r‐CMRO (regional cerebral metabolic rate of oxygen), and local cerebral oxygen metabolism rate [22, 23]. This phenomenon has been described as a sympathetic arousal response, although that does not directly induce consciousness recovery.

In the CAN model, the ANS is involved in the modulation of autonomic outputs in response to pain, emotional, and behavioral stimuli. These autonomic regulatory structures are functionally highly correlated with cardiac function [18, 24, 25]. Riganello et al. conducted a quantitative assessment of the markers associated with the CAN. Variations in the mean heart rate within a fixed time window reflect the regulatory function of the circulatory control system and are now considered reliable indicators of the interaction between sympathetic and parasympathetic functions and their influence on cardiac function [26, 27]. Hence, heart rate variability (HRV) measurements are deemed to reflect ANS dynamics and cardio‐cerebral interactions, potentially indirectly linking to higher‐order brain functions [18, 21, 28, 29]. Within this context, measures of cardiac functional stability and variability can be found in HRV assessments (i.e., variables analyzed in time‐domain, frequency‐domain, and nonlinear measurements), reflecting the heart's resilience in adapting to ever‐evolving circumstances [19, 30, 31]. Wijnen et al. utilized HRV analysis to demonstrate a positive correlation between the level of consciousness and ANS modulation in patients with DOC, highlighting the potential for clinical assessment and consciousnesses stratification [32]. Riganello et al. conducted a quantitative evaluation of HRV in DOC patients, revealing its diagnostic stratification capabilities for DOC and prognostic assessment, as evidenced by the grading of brain function at 3 months via telephonic assessment [33, 34]. In a study on HRV responsiveness to auditory stimuli, Raimondo also found that, compared to patients with VS/UWS, patients with MCS exhibited no difference in baseline cardiac activity and spectral power of HRV at rest. However, during auditory stimulus‐related tasks, MCS patients demonstrated shorter intervals between R peaks [35].

Given that individual indices of HRV have been examined independently in previous studies, there may exist interactions and significant multicollinearity among them. Therefore, machine learning approaches become crucial, as they are capable of leveraging multiple data sources related to clinical disease characteristics and the variations in pertinent research indicators. Consequently, by tracking the clinical outcomes of patients, we aim to explore the potential role of HRV‐derived components in improving consciousness status, thereby assessing the prognostic predictive capacity of DBS treatment. Currently, there is a lack of research investigating the characteristic changes in HRV under the modulation of central thalamic DBS. Therefore, the aim of this study is to examine whether HRV‐derived indices exhibit consistent response patterns related to DBS stimulation in patients with DOC undergoing CT‐DBS treatment, across different stimulation parameters, and to identify HRV‐derived indices that are closely correlated with clinical outcomes. Such research may empower clinicians to effectively implement neuromodulation therapies for patients with DOC based on early indicators of changes in their electrocardiographic (ECG) signals.

## 2 Methods

### 2.1 Patient Enrollment

**FIGURE 1:**
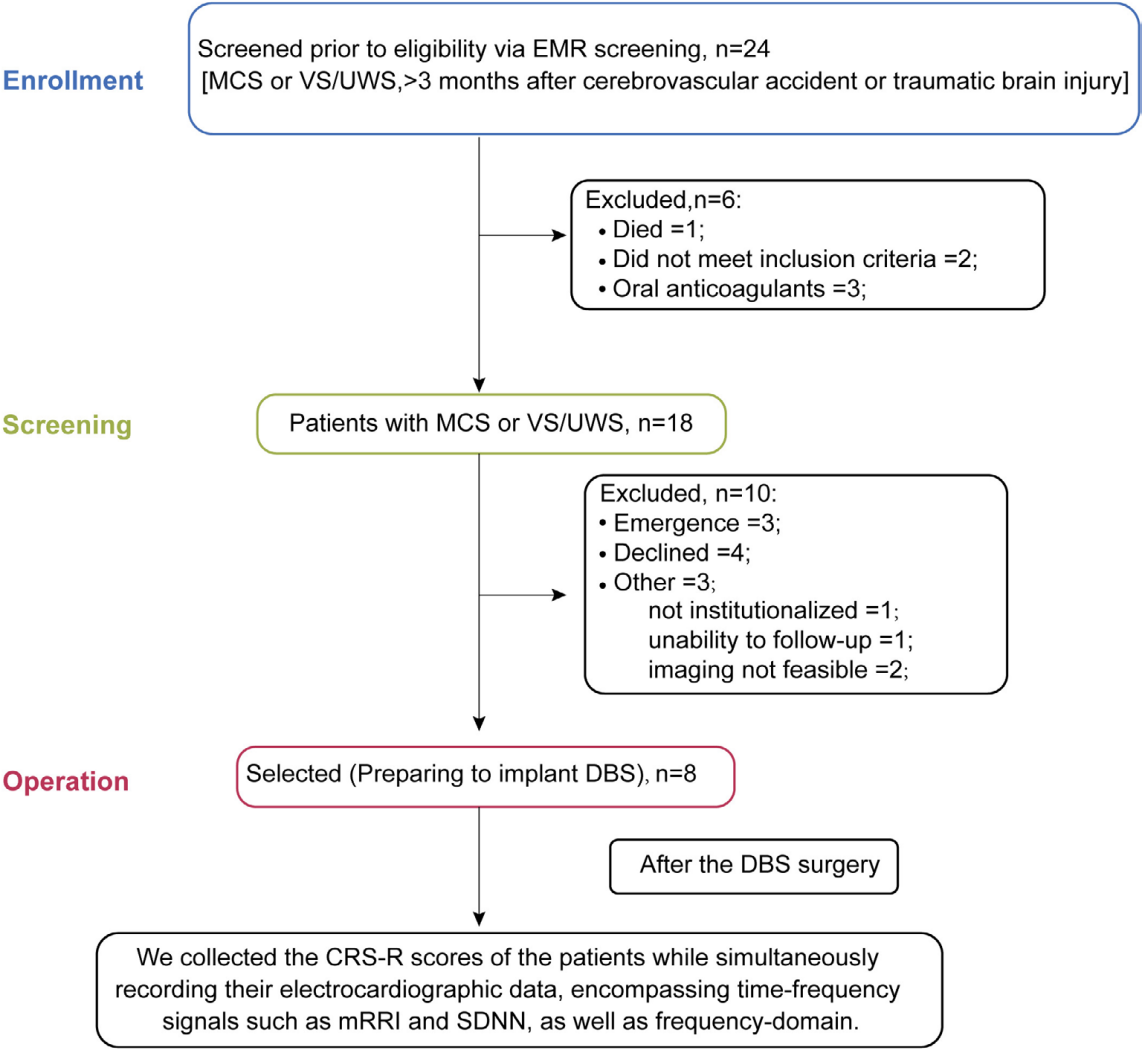
Illustration of the process of patient inclusion, which involved reviewing EMR. Initially, a total of 24 patients were screened. However, one patient died during hospitalization and two patients met the exclusion criteria. Additionally, three patients were already receiving anticoagulant therapy. Among the remaining 18 eligible candidates, three patients experienced emergencies, four family members refused to participate, one patient were unable to return for hospital surgery, one patient could not be followed up on, and two patients showed severe brain damage on imaging examinations. Consequently, eight patients were ultimately included in the DBS surgical procedure. After the DBS surgery, we collected all the patients' clinic statistic. CRS‐R, JFK Coma Recovery Scale‐Revised; DBS, Deep brain stimulation; EMR, Electronic medical records; HF, High frequency; LF, Low frequency; MCS, Minimally conscious state; mRRI, Mean of the R‐R intervals; SDNN, Standard deviation of normal‐to‐normal intervals; TP, Total power; VS/UWS, Vegetative state/unresponsive wakefulness syndrome.

We screened DOC patients from August 2022 to December 2023 at Beijing Tiantan Hospital and enrolled those scheduled for DBS surgery into our study. The inclusion criteria were as follows: (a) male or female patients aged between 15 and 80 years old; (b) diagnosis of DOC, including VS/UWS or MCS, confirmed by CRS‐R assessment; (c) onset of DOC more than 3 months prior to enrollment; (d) known causes of DOC, such as TBI, congenital heart disease, or hypoxic–ischemic encephalopathy. The exclusion criteria are as follows: (a) patients with severe cardiovascular, digestive, renal, hematological, endocrine, respiratory diseases, immunodeficiency, tumors, or other severe illnesses; (b) patients who have undergone major surgery within the last 3 months or have received experimental drug treatment with uncertain safety within the last 1 month. This study was conducted in accordance with the latest version of the Helsinki Declaration, and the protocol was approved by the Ethics Committee of Beijing Tiantan Hospital (Approval Number: KY2023‐161‐03) and registered with the Chinese Clinical Trial Registry (Registration Number: ChiCTR2400085855). All patients participating in this study were covered by clinical trial insurance. Figure 1 illustrates the process of patient enrollment and screening. Ultimately, only eight patients' guardians consented to DBS implantation surgery.

### 2.2 DBS Surgery

Patients were placed under general anesthesia for the installation of the stereotactic base ring. Axial, coronal, and sagittal T1‐weighted MRI sequences were performed after the placement of the Leksell G frame (Elekta, Sweden) with a slice thickness of 2 mm. The electronic atlas of the thalamic nuclei was superimposed in the surgical planning system to enhance intraoperative localization accuracy. A quadripolar electrode (L302, PINS, China) was implanted into the bilateral central thalamic. The implantable pulse generator (IPG, G102RZ, PINS, China) was placed at the midpoint of an imaginary line between the subclavian and anterior axillary lines and the middle sternal line. Postoperative CT or 1.5T MRI scans were performed to confirm the implantation location.

### 2.3 Experimental Design and Data Collection

**FIGURE 2:**
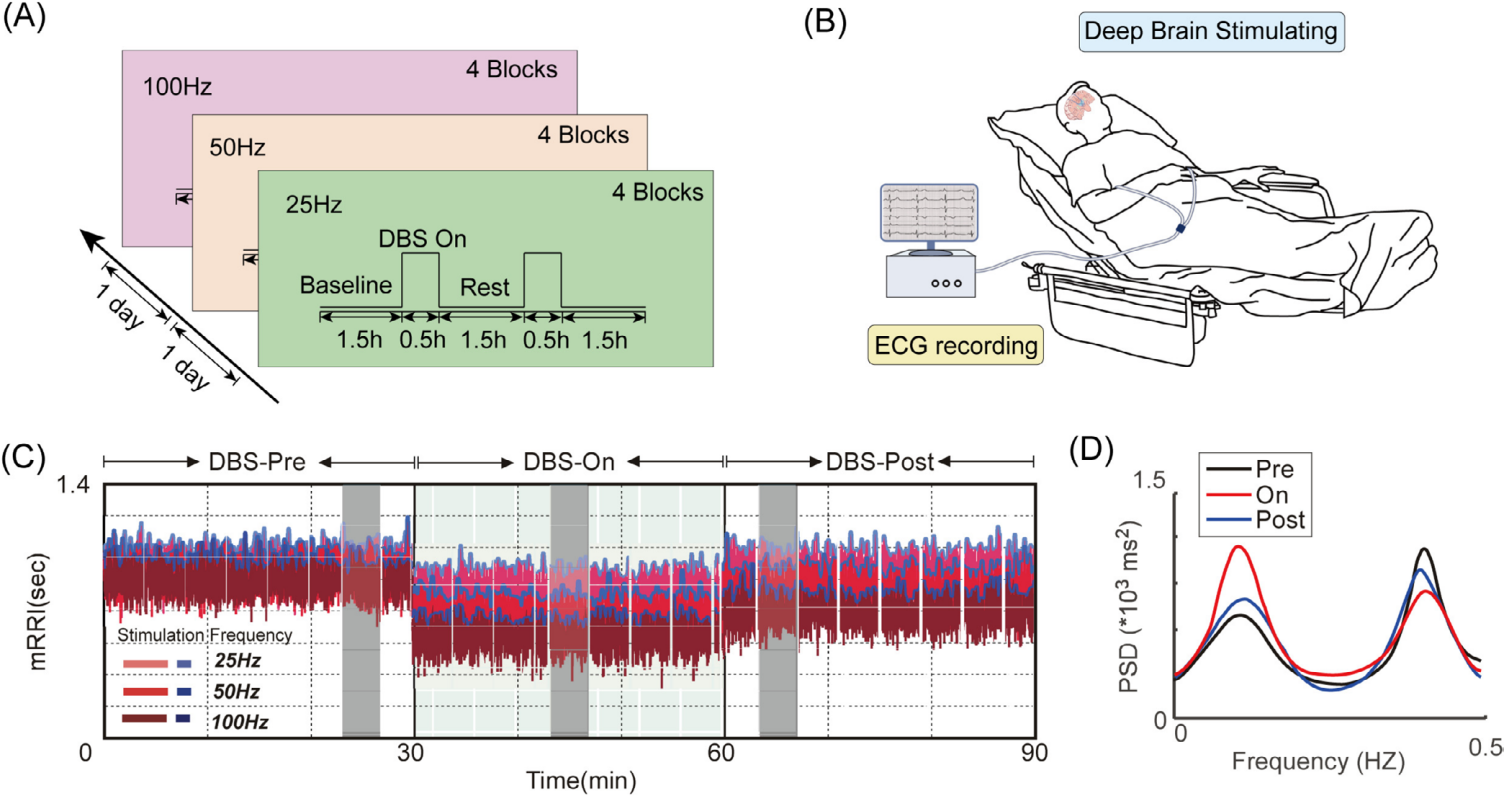
(A) Experimental paradigm illustrating the application of DBS at different frequencies (25 Hz, 50 Hz, and 100 Hz) for 1 day, with four blocks per day. Each block consisted of 30 min of stimulation followed by 90 min of rest. (B) Diagram showing the collection of ECG signals through disc electrodes placed on the mid‐axillary lines of both sides of the patient during the stimulation period. The ECG data of each patient were used for HRV indices analysis. (C, D) Analysis of time‐domain and frequency‐domain of HRV in Patient P3. (C) The time‐frequency plot of processed ECG signals during the experiment, with gray boxes indicating three 3‐min periods before (Ph1: DBS‐Pre), during (Ph2: DBS‐On), and after (Ph3: DBS‐Post) stimulation. mRRI derived from ECG signals obtained under three distinct DBS stimulation frequencies. The blue curve represents the envelope curve of mRRI. (D) The frequency‐domain curves across the three phases reveal instantaneous effects observed in Patient P3, characterized by an elevation in LF components during DBS‐On phase, followed by a subsequent decrease during DBS‐Post phase (15‐s fragment). DBS, Deep brain stimulation; ECG, Electrocardiogram; mRRI, Mean of the R‐R intervals; PSD, Power spectral density.

Following the initial week of undergoing DBS, after extensive titration tests on stimulation frequency and intensity, the optimal geometry of each electrode contact was determined based on the limitation of significant arousal effects and visible side effects. Subsequently, all patients underwent sequential stimulation with Group A, Group B, and Group C, with each group receiving stimulation for 1 day. The DBS stimulation was turned on for 30 min, followed by 90 min of DBS‐off time during daylight hours (10:00 AM to 18:00 PM). The stimulation parameters for Group A were set at a frequency of 25 Hz, voltage of 3.0 V, and pulse width of 120 μs. For Group B, the parameters were 50 Hz frequency, 3.0 V voltage, and 120 μs pulse width. Lastly, Group C utilized a frequency of 100 Hz, voltage of 3.0 V, and pulse width of 120 μs (see Figure 2A). During the stimulation periods, the ECG were collected through disc electrodes attached to the mid‐axillary lines on both sides of the patient (see Figure 2B). The data were recorded using the high‐density ECG acquisition software NSH‐R (Version 1.00.00.9742, Neuracle, China) with a sampling rate of 16 kHz and a 50 Hz filter applied. Demographic characteristics related to DOC (age, gender, etiology, duration of illness, CRS‐R score, clinical diagnosis), as well as changes in CRS‐R scores post‐DBS stimulation, were recorded and monitored continuously for up to 6 months. After admission, two experienced neurosurgeons conducted CRS‐R assessments (at least four times per week) for each patient, scheduled between 8:00 AM and 10:00 AM to minimize the impact of arousal fluctuations and reduce the risk of misdiagnosis.

During the follow‐up period, we titrated the DBS parameters with reference to clinically observed adverse effects such as muscle rigidity, strabismus, and emotional changes. Additionally, we documented changes in the CRS‐R scores of all the patients over a 6‐month period.

### 2.4 HRV and HRV‐Related Metrics

In accordance with the guidelines of the European Society of Cardiology and the North American Society of Pacing and Electrophysiology, the quantitative HRV analysis in this study was conducted as follows [27]. The initial raw ECG data was extracted using the NSH‐R data acquisition software program. Three‐minute segments immediately before, during, and after each DBS stimulation, with no significant movement artifacts, were selected for each group, while ensuring that there were no severe disturbances and ectopic beats.

The RR interval (RRI) sequencies extracted from the original ECG signal by bandpass filtering based on a second‐order difference RR interval algorithm, which is used to calculate common short‐range time‐domain parameters, including mean heart rate interval (mRRI) and standard deviation of normal‐to‐normal intervals (SDNN). A quadric spline resampling process of 4 Hz was performed on the RRI to obtain a uniformly sampled time series. Then, smoothing prior principle was adopted to remove the trend term component of ultra‐low frequency, and 19‐order AR model power spectrum estimation based on Burg algorithm was adopted to calculate the frequency‐domain parameters of HRV.

Frequency‐domain parameters include: Total power (TP) in the selected frequency range (0.04–0.4 Hz) reflects the total activity of the autonomic nervous system; HF power in the range of high frequency (0.15–0.4 Hz) reflects the activity of vagus nerve; LF power in the low‐frequency range (0.04–0.15 Hz) mainly reflects the activity of the sympathetic nerve [27, 36, 37]. HFnorm calculated by HF/(LF + HF) * 100 reflects the relative value of vagus nerve activity; LFnorm calculated by LF/(LF + HF) * 100 reflects the relative value of sympathetic nerve activity. The low‐frequency to high‐frequency power ratio LF/HF is the quantification of the balance between the two [13, 38]. These parameters reflect the activity and balance of the ANS [14, 39].

### 2.5 Statistical‐Based Feature Selection

Firstly, we prepare the DBS‐pre and DBS‐post's difference values for each individual's HRV indicators across three different stimulation frequencies. Subsequently, based on different prognoses (outcome), we categorize the data into two groups, forming the original dataset. Then, we employ the SHapley Additive exPlanations (SHAP) methodology, which is designed to offer a fair allocation of the contribution of each feature toward the model's predictions, to analyze the weight of all these features and select the key features influencing patient classification [40, 41]. Finally, utilizing an support vector machine (SVM) classifier and based on five‐fold cross‐validation, we randomly divide both the full set of features and the selected features into five equally sized subsets for training and validation [42]. Cross‐validation is a widely used method to assess the performance of a model by dividing the data into multiple subsets and training the model on each subset while validating it against the remaining data. Five‐fold cross‐validation is a robust method for assessing the performance of a machine‐learning model. In this procedure, the original dataset is randomly partitioned into five equal‐sized subsets or “folds”. Each of these subsets comprises approximately 20% of the data. The model is then trained and validated five times, each time using a different subset as the validation set, and the remaining 80% of the data (the other four subsets) as the training set. In this method, each individual data point is included in the validation set one time only and contributes to the training set in four out of the five iterations. After these iterations, the model's performance is computed by taking the average across all five rounds of validation [43].

### 2.6 Statistical Analysis

Statistical analysis was performed using SPSS software version 23 (IBM, USA). Descriptive data were presented as means and standard deviations for continuous variables and counts and percentages for categorical variables, with histograms or boxplots used to visually assess data distribution. The Mann–Whitney test and Chi‐square analysis were employed for numerical and categorical variables, respectively. Normality tests were conducted on the ECG data, and repeated measures ANOVA were used for cross‐effects before and after each stimulus parameters. A *t*‐test was performed to compare changes in characteristic parameters between groups before and after the frequency modulation trial. Changes in CRS‐R scores at 6‐month follow‐up were used as independent variables to assess HRV dependent variables related to consciousness level improvement. A *p*‐value < 0.05 was considered statistically significant.

## 3 Results

### 3.1 Clinical Characteristics

A total of eight DOC patients who met the inclusion criteria underwent DBS surgery. The mean age of all was 47.5 ± 15.41 years, with seven males and one female. Preoperatively, five patients were diagnosed with VS/UWS (with CRS‐R total scores of 5, 7, 7, 8, and 8), while 3 patients were diagnosed with MCS (with CRS‐R total scores of 9, 9, and 10). The etiologies of DOC included trauma (*n* = 2), brainstem infarction (*n* = 4), and hypoxic–ischemic encephalopathy (*n* = 2). The time elapsed from the onset of DOC to surgery was 7.25 ± 2.71 months, as detailed in Table 1.

**TABLE 1:** Clinical characteristics and deep brain stimulation (DBS) target information of 8 patients with disorders of consciousness (DOC).

Patient	Age	Gender	Etiology	Duration of stable DOC (months)	CRS‐R total score	Clinical Diagnosis	DBS target
1	32	M	Brainstem infarction	11	7 (112102)	VS	Bilateral central thalamic
2	51	M	Brainstem infarction	7	8 (221102)	VS	Bilateral central thalamic
3	45	F	Brainstem infarction	8	8 (122102)	VS	Bilateral central thalamic
4	51	M	Anoxia	10	10 (223102)	MCS−	Bilateral central thalamic
5	19	M	Anoxia	7	9 (123102)	MCS−	Bilateral central thalamic
6	66	M	Trauma	4	9 (132102)	MCS−	Bilateral central thalamic
7	55	M	Trauma	8	7 (112102)	VS	Bilateral central thalamic
8	61	M	Brainstem infarction	3	5 (110102)	VS	Bilateral central thalamic
Mean ± SD	47.50 ± 15.41			7.25 ± 2.71	7.88 ± 1.55		

Abbreviations: CRS‐R, JFK coma recovery scale‐revised; F, female; M, male; MCS−, minimally conscious state minus; VS, vegetative state.

### 3.2 Acute Effects of DBS Stimulation

We analyzed changes in time‐domain and frequency‐domain of HR during DBS‐Pre, DBS‐On and DBS‐Post to evaluate the acute effects of DBS. In the context of patient P3, Figure 2C,D illustrate the changes in mRRI in DBS‐Pre, DBS‐On and DBS‐Post phase with different stimulation parameters, as well as the average changes in the power spectrum of ECG signals. The results demonstrate during the DBS‐On phase a reduction in the time‐domain index, mRRI, accompanied by a significant increase in frequency‐domain indices, particularly in the LF band.

**FIGURE 3:**
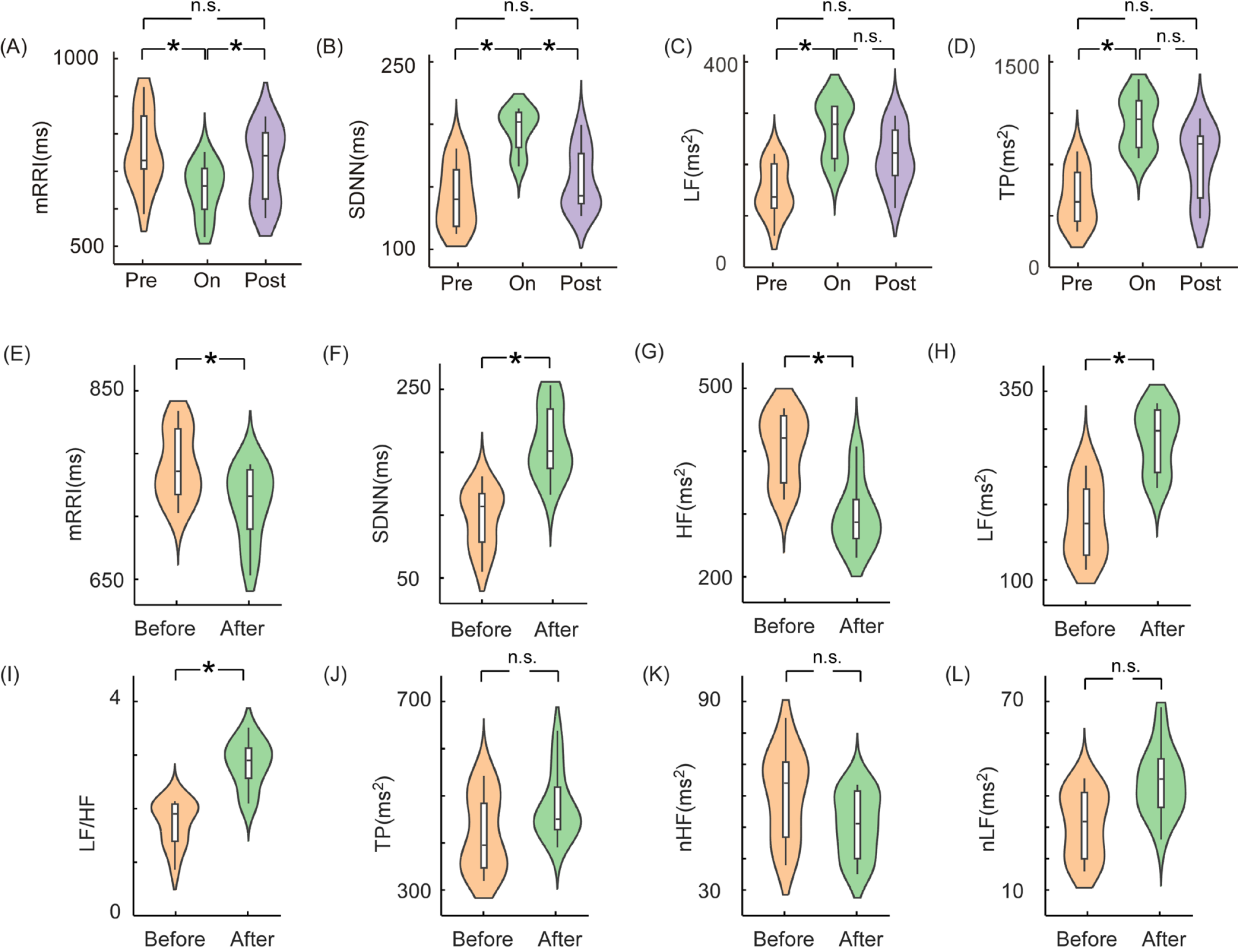
We calculated the average values across all stimulation frequency groups over these 3 days to emphasize the differences observed Pre, On, and Post the application of DBS stimulation. Instantaneous changes in time‐domain indices, mRRI (A) and SDNN (B), before and after the activation of DBS in all patients revealed significant alterations during the DBS‐On phase, and returned to normal once the DBS was deactivated. Similarly, frequency‐domain indices, LF (C) and TP (D), also demonstrated prominent changes during the DBS‐On phase, failing to return to baseline levels when the DBS was deactivated. After 3 days of temporary stimulation, the HRV results of all patients indicated a significant decrease in mRRI (E) and HF (G), accompanied by a notable increase in SDNN (F), LF (H), and LF/HF (I). However, no statistically significant differences were observed in the changes of TP (J), nHF (K), and nLF (L). “Before” refers to the baseline period before the entire 3‐day experiment, while “After” refers to the rest period following the conclusion of the entire 3‐day experiment. (*p < 0.05, n.s. *p* ≥ 0.05). HF, high frequency; HRV, heart rate variability; LF/HF, low to high‐frequency ratio; LF, low frequency; mRRI, mean R‐R interval; nHF, normalized high frequency; nLF, normalized low frequency; SDNN, standard deviation of normal‐to‐normal intervals; TP, total power.

We conducted a detailed analysis of time‐domain and time‐frequency indices in DBS‐Pre, DBS‐On and DBS‐Post phase of all the patients. Figure 3A illustrates the time‐domain results, revealing a significant decrease in the mRRI during the DBS‐on phase (*p* = 0.012, *η* ^2^ = 0.27), followed by a rapid return to baseline during the DBS‐post phase (*p* = 0.018, *η* ^2^ = 0.42). Conversely, Figure 3B demonstrates a notable increase in SDNN during the DBS‐on period (*p* = 0.044, *η* ^2^ = 0.31), which subsequently declines rapidly to baseline in the DBS‐post phase (*p* = 0.0.011, *η* ^2^ = 0.42). The analysis of frequency‐domain indices reveals intriguing patterns. Specifically, Figure 3C shows a marked increase in LF power during the DBS‐on period (*p* = 0.038, *η* ^2^ = 0.26). While LF power exhibited a relatively decline in DBS‐Post period, but this decline did not statistically significance. Similarly, Figure 3D demonstrates a significant elevation in TP during the DBS‐on phase (*p* = 0.041, *η* ^2^ = 0.18). Following DBS‐on, there was a decline in TP, yet the change was not statistically significant (See Tables S1 and S2).

### 3.3 Short‐Term Changes in HRV Indices Following Central Thalamic Stimulation

After the completion of the 3‐day temporary stimulation period, the time‐domain and frequency‐domain components of HRV were analyzed. The results indicated that all patients experienced a significant decrease in the time‐domain index mRRI (*p* = 0.024, Figure 3E) and a marked increase in SDNN (*p* = 0.018, Figure 3F) following DBS stimulation. Frequency‐domain analysis revealed a significant reduction in HF (*p* = 0.025, Figure 3G) and a substantial increase in LF (*p* = 0.01, Figure 3H), with the LF/HF ratio also displaying a significant elevation (*p* = 0.031, Figure 3I). Conversely, TP, nHF, and nLF did not show notable changes (Figure 3J–L). These short‐term variations in HRV demonstrate the cumulative effect of central thalamic DBS‐on the stimulation of the ANS (See Table S3).

### 3.4 Characteristics of HRV Changes Induced by Different Frequencies

**FIGURE 4:**
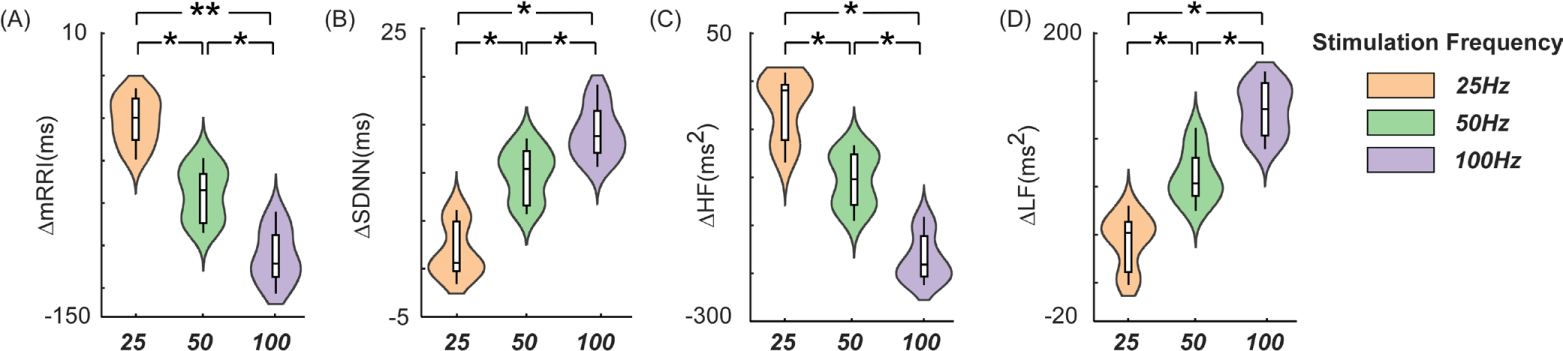
The result illustrates the statistical results of the differences in HRV indices between DBS‐pre and DBS‐post for different stimulation frequency groups (25, 50 and 100 Hz). The time‐domain indices show a decrease in ΔmRRI (A) and an increase in ΔSDNN (B), while the frequency‐domain indices indicate a decrease in ΔHF (C) and an increase in ΔLF (D). (*p < 0.05, n.s. *p* ≥ 0.05). HF, high frequency; LF, low frequency; mRRI, mean R‐R interval; SDNN, Standard deviation of normal‐to‐normal intervals.

We calculated the differences in HRV indices between DBS‐pre and DBS‐post for each stimulation frequency group and performed statistical analysis on these differences to observe the variations in HRV induced by different stimulation parameters. As shown in Figure 4A, for the time‐domain index mRRI, significant decreases were observed in the 50 Hz and 100 Hz stimulation groups compared to the 25 Hz stimulation group (*t* = 3.3, *p* = 0.024; *t* = 4.2, *p* = 0.011, respectively). Figure 4B demonstrates an increase in SDNN across all three stimulation groups, with significant elevations in the 50 Hz and 100 Hz groups compared to the 25 Hz group (*t* = 2.4, *p* = 0.034; *t* = 3.4, *p* = 0.042, respectively). Figure 4C presents the frequency‐domain results, showing a decrease in HF for both 50 Hz and 100 Hz compared to 25 Hz (*t* = 3.4, *p* = 0.026; *t* = 2.7, *p* = 0.044, respectively). Meanwhile, Figure 4D indicates a pronounced increase in LF components for 50 Hz and 100 Hz compared to 25 Hz (*t* = 4.5, *p* = 0.025; *t* = 4.3, *p* = 0.014, respectively).

### 3.5 Correlation Between CRS‐R Improvement and Changes in HRV after 6 Months Follow‐Up

**FIGURE 5:**
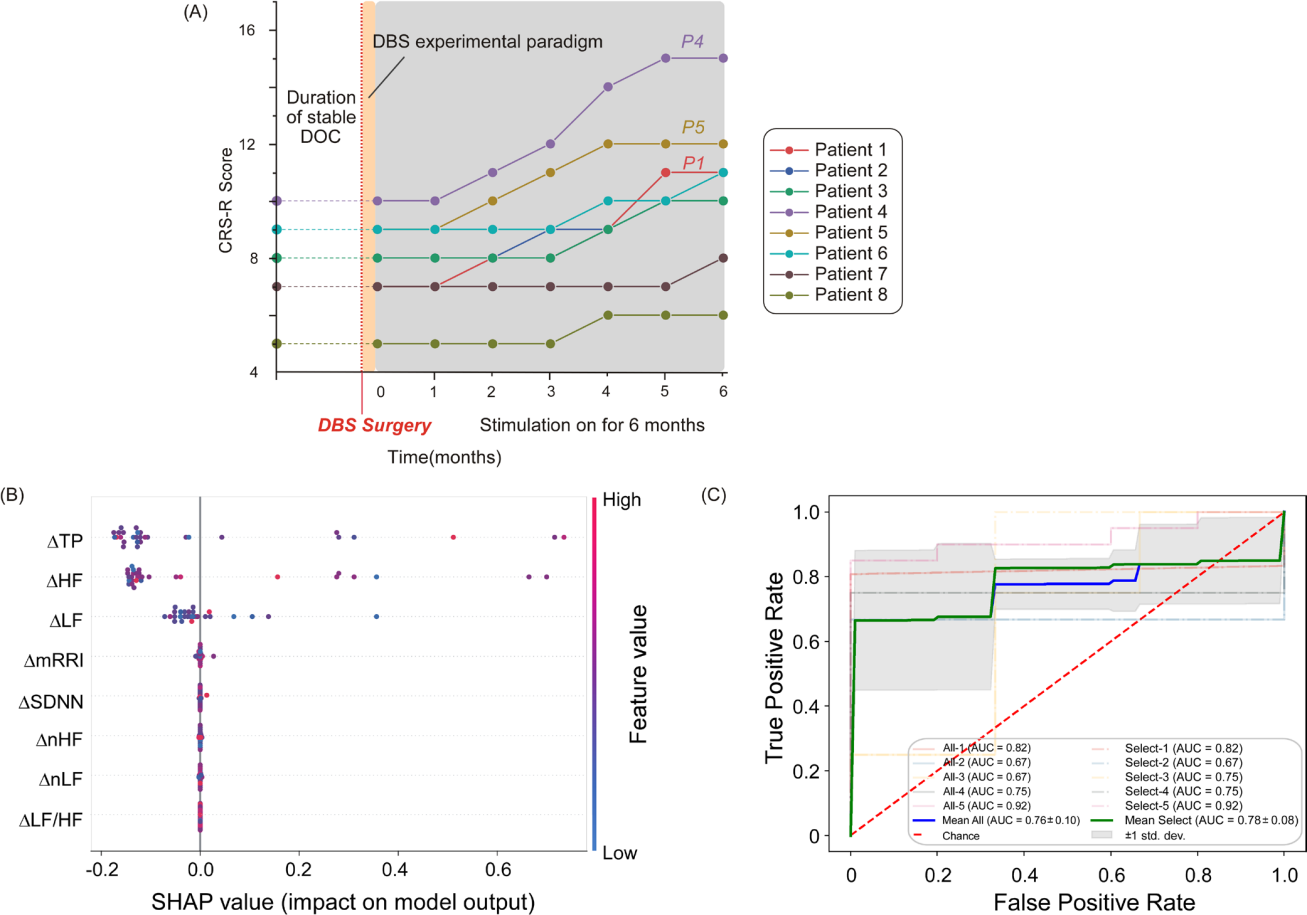
(A) CRS‐R scores of all patients before and after DBS surgery. The red bold line indicates the time of DBS surgery, the orange area represents the period of experimental paradigm for short‐term DBS stimulation, and the gray shaded area represents the continuous DBS stimulation period. The follow‐up last for 6 months. At the 6‐month follow‐up after initiating DBS, three patients (P1, P4, and P5) showed an improvement of more than 3 points in their CRS‐R scores, indicating effective stimulation (An increase in CRS‐R score of more than 3 points were considered as significant responses to stimulation). (B) Depicts the classification of patients into two outcome groups (improved vs. unimproved), utilizing the SHAP methodology to analyze the relative importance or weight of eight HRV indices. (C) Showcases that leveraging the top three features (TP, HF, and LF), yields classification results that are in agreement with those derived from the entire feature set. AUC, area under curve; CRS‐R, JFK Coma Recovery Scale‐Revised; DBS, deep brain stimulation; DOC, disorder of consciousness; HF, high frequency; LF, low frequency; mRRI, mean of the R‐R intervals; nHF, HFnorm; nLF, LFnorm; PSD, Power spectral density; SDNN, standard deviation of normal‐to‐normal intervals; SHAP, SHapley additive exPlanations; TP, total power.

After 6 months, three patients (P1, P4, and P5) demonstrated an increase in CRS‐R scores of three points or more, indicating significant effectiveness of the stimulation. The remaining five patients had CRS‐R score improvements of less than three points, considered as ineffective stimulation, as shown in Figure 5A. The results indicate that in the group of patients deemed to have responded positively to the stimulation, statistically significant differences were observed in the changes of LF, HF, and TP within HRV (*p* < 0.05). In contrast, no statistically significant differences were found in the changes of mRRI, SDNN, nHF, nLF, and LF/HF (*p* > 0.05, See Table S4).

Subsequently, we employed those various features changes in DBS‐on and DBS‐post phase including TP, HF, LF, HF/LF, nHF, nLF, mRRI, and SDNN to classify the two groups (improved vs. unimproved). The SHAP method was utilized to analyze the weights of the aforementioned features, which depicted in Figure 5B. Among these indicators, the first three—TP, HF, and LF—exhibit a significant impact on the model and are considered pivotal factors influencing patient classification (See Figure S1). This finding aligns with the results of our aforementioned statistical analysis in Table S4. Subsequently, we classified patients based solely on these three features and compared the outcomes with those derived from classifications using all features, as depicted in Figure 5C. The “mean” value represents the average outcome across the five folds cross‐validation. The results indicate that utilizing the three selected features (AUC = 0.78 ± 0.08) yields a slightly higher classification performance compared to using all features (0.76 ± 0.10). This suggests that the selected three features are representative of the entire feature set. Furthermore, the variance observed for the selected features is lower than that for all features, indicating that the classification performance of these three features is more stable and that redundant features have been reduced. The classifier utilized in this experiment was the SVM, and the classification results were based on five‐fold cross‐validation.

## 4 Discussion

Due to the uncertainty surrounding the pathological mechanisms underlying DOC, current therapeutic approaches are continually under exploration [44]. As indicated by existing literature reviews, there remains some controversy regarding the effective stimulation parameters for DBS [45]. Schiff et al. suggests that high‐frequency stimulation, specifically at 100 Hz, can enhance patients' behavioral responsiveness, potentially by strengthening prefrontal cortex connectivity [10]. Our recent clinical, controlled, retrospective study indicates that 100 Hz DBS stimulation is conducive to improving patients' level of consciousness 1‐year post‐treatment, with overall improvement in consciousness at 1 year achieved in 32.4% [7]. In contrast, recent work by Arnts et al. using 50 Hz stimulation found potentially better outcomes than those achieved with 130 Hz. Electroencephalogram (EEG) results demonstrated an enhancement of relevant brain functional network connections across all frequency bands [46]. Meanwhile, a 10 years single‐center study by Chudy et al. suggests that low‐frequency stimulation (25–40 Hz) is more commonly used than high‐frequency stimulation. However, the benefits of using either low or high frequencies remain unclear [47]. It is recommended that neurophysiological testing be incorporated as part of the selection criteria for DBS candidates and can also serve as a relatively reliable method for predicting the prognosis of patients with DOC. In summary, the selection of proper stimulation frequencies for DBS currently necessitates a synthesis of clinical experience, the specific condition of the patient, and existing research evidence for each individual patient. Consequently, our study has selected three stimulation parameters of 25 Hz, 50 Hz, and 100 Hz to investigate HRV in the context of exploring the impact on DOC.

The CAN is an integral component of an internal regulation system through which the brain receives viscerosensory inputs (relayed on the nucleus of the tractus solitarius) and humoral inputs (relayed through the circumventricular organs), and controls sympathetic and parasympathetic activities, neuroendocrine and other essential activities for survival [48, 49]. It encompasses the cerebral cortex (especially insular cortex), amygdala, hypothalamus (especially paraventricular nucleus, PV), autonomic nuclei in the brain stem (especially rostral ventrolateral medulla, nucleus of the tractus solitarius, periaqueductal gray matter and parabrachial complex), and the lateral horn of the spinal cord [48, 50]. The top‐down output of the CAN modulates the ANS via the stellate ganglion and the vagus nerve. The CAN output regulates vagal reflexes through GABAergic neurons located in the NTS, thereby exerting primary inhibitory control over the heart [51, 52].

Thus, the potential underlying cause of DOC may involve damage to the CAN, leading to a decline or loss of ANS regulation. Cai et al. demonstrates that patients with DOC exhibit ANS dysfunction and neuropathy, with significant delays and reductions in their autonomic responses throughout the head‐up tilt (HUT) test [28]. Riganello et al. further distinguish different levels of consciousness based on the extent of decline in autonomic regulation among DOC patients [14, 19]. The interplay between the CNS and the ANS may serve as a potential mechanism underlying the autonomic nervous dysfunction accompanying DOC.

Our study found that after the initiation of DBS, under different stimulation conditions, significant changes occurred in the time‐domain indices of mRRI and SDNN, as well as the frequency‐domain related indices of HF and LF. The acute stimulatory response induced by DBS is reversible, with HRV indices returning to baseline after stimulation cessation. In fact, this observation aligns with clinical phenomena where stronger stimulation parameters during the postoperative titration phase often elicit sympathetic excitation, necessitating a reduction in stimulation intensity. This is consistent with findings reported by Yamamoto, Tsubokawa, and Arnts [22, 23, 46]. These acute‐phase effects consistently manifest with stimulation and dissipate upon its cessation, failing to reflect genuine changes in patient consciousness.

Interestingly, across the three sets of different stimulation parameters, it was consistently observed that 25 Hz produced the smallest stimulatory effect, while 100 Hz elicited the largest. This variation manifested as a decrease in mRRI and HF, and an increase in SDNN and LF, with increasing stimulation frequency. As summarized in a series of publications by Riganello [12, 14, 33, 34], the functional contributions of the ANS can be distinguished through frequency‐domain analysis, typically categorized into three bands: HF (0.15–0.5 Hz), LF (0.04–0.15 Hz), and very low frequency (VLF) (0.0033–0.04 Hz). The power, relative power, and the ratio of HF to LF (LF/HF) are commonly calculated parameters. HF reflects parasympathetic activity, specifically heart rate variations associated with the respiratory cycle [53]. LF primarily reflects baroreceptor activity at rest, also known as the ‘baroreceptor range’ [54]. The physiological mechanisms underlying the VLF band remain unclear. The interpretation of the LF/HF ratio is controversial, with LF power potentially arising from sympathetic activity and HF power from parasympathetic activity. An increased LF/HF ratio indicates dominance of either the parasympathetic or sympathetic nervous system, leading to a similar trend in time‐domain indices and instantaneous responses, manifested as a sympathetic excitation response [12, 14]. Therefore, at 100 Hz, patients exhibited a more pronounced sympathetic excitation response, consistent with changes in the time‐domain. This observation aligns with Schiff's findings, where patients experienced acute arousal changes under 100 Hz stimulation, including accelerated heart rate, good eye opening, and sound pursuit [10]. These results were verified in all patients after 3 days of short‐term stimulation. In summary, DBS induced positive changes in HRV time‐domain indices, accompanied by increases in LF and LF/HF components and decreases in HF in the frequency‐domain.

In the autonomic regulation of the CAN, the thalamus serves as a pivotal top‐down regulatory structure, while extensive research has discovered its role in arousal and consciousness. Specifically, in rodents, the midline nuclei of the thalamus are divided into two major categories along the dorsoventral axis: the dorsal midline nuclei, which comprise the PV and the parathalamic nucleus (PT). In recent years, the PV nucleus has emerged as a highly interconnected node within the brain networks responsible for sensing and regulating homeostatic behaviors [55, 56]. Studies in animals have revealed that PV nucleus participate in the regulation of arousal and are associated with increased arousal and exploratory behavior in mice [55]. The interaction between the thalamus and central autonomic control mechanisms is poorly understood. In the disease of fatal familial insomnia (FFI), patients exhibit significant abnormalities in the autonomic and circadian. FFI is recognized as a “preferential thalamic degeneration” in which the anteroventral (AV) and mediodorsal (MD) thalamic nuclei are invariably affected by a precocious and large cell loss [57]. Notably, the medial segment of the MD nucleus is adjacent to the PV nucleus, and the medial portion of the MD may share relay functions with the adjacent PV, in circuits involving the medial prefrontal cortex, amygdala, and dorsomedial hypothalamus, thereby controlling autonomic functions [58, 59]. Consequently, this may explain the loss of normal autonomic regulation by the damaged MD and PV nuclei within the thalamus of FFI patients, leading to elevated arterial pressure and heart rate compared to healthy individuals [59]. In this study, we investigated the arousal‐facilitating potential of DBS targeted at the central thalamus in DOC, while concurrently observing the modulatory effects of DBS‐on the ANS. It is possible that DBS, by stimulating the surrounding regions of the central thalamus, may contribute to influencing the function of PV nucleus, thereby modulating changes in HRV.

Currently, the most significant application of HRV in patients with DOC is to differentiate between patients in VS and MCS [13, 19, 28, 30]. In the final section of our work, the results support the hypothesis that HRV is correlated with predicting the improvement of consciousness in patients with DOC following DBS. In summary, during our long‐term follow‐up of patients with DBS, we observed an increase in the LF and TP components of HRV and a decrease in the HF component. This finding indicates that these patients retain greater reactivity within the central autonomic neural network (CAN‐ANS), enabling them to adjust heart rate variations in response to prolonged DBS neuromodulation [26]. The results indicate an increase in sympathetic activity and a decrease in parasympathetic activity following stimulation. However, when considering these three parameters collectively, the enhanced activity of the autonomic nervous system is correlated with the patients' long‐term recovery.

Furthermore, we employed SHAP values to quantify the marginal contribution of eight features within HRV to the prediction outcomes. We have estimated the top three strongest predictors to be TP, HF, and LF. From the perspective of physiological mechanisms, the low‐frequency band (0.04–0.15 Hz) is generally associated with sympathetic activity, whereas the high‐frequency band (0.15–0.40 Hz) is correlated with parasympathetic activity [53, 60]. From the statistical results, it appears that DBS may enhance HRV in patients with favorable prognoses by upregulating sympathetic activity and downregulating parasympathetic activity. Conversely, patients exhibiting lower HRV in response to DBS stimulation tend to have poorer prognoses. We conducted a five‐fold cross‐validation to compare the predictive performance of the top three strongest indicators with that of the overall set of indicators. The results indicate that the area under the curve (AUC) for our estimated top three predictors is higher than that for the model incorporating all features (0.78 vs. 0.76), indicating that TP, HF, and LF can effectively represent the overall HRV indices in predicting the long‐term prognosis of patients with DOC.

Therefore, this study represents the first observation of ANS functional activity beyond the targeted effects of DBS of the central thalamus in DOC. Notably, we found that ANS activity is modulated by the frequency of stimulation, with increased stimulation frequency leading to a more pronounced ANS response, manifested as elevated HRV. This finding suggests a potential mechanism by which the modulation of thalamo‐cortical and thalamo‐striatal neural networks can influence the function of these pathways [61]. Specifically, CT‐DBS, by enhancing thalamo‐cortical connectivity, may indirectly regular the thalamus's capacity to integrate signals from the autonomic nervous system, thereby influencing HRV, [1, 7, 62, 63, 64, 65] especially in the patients with a good prognosis or prognosis potential. Another significant finding of this study underscores the potential of changes in frequency‐domain indices of HRV as biomarkers for predicting the prognosis of patients with DOC. This observation emphasizes the necessity of developing therapeutic interventions aimed at restoring the sympathovagal balance, which may ultimately improve the clinical prognosis of DOC patients.

There have some limitations. Firstly, our cohort included only eight patients who underwent DBS surgery, resulting in a relatively small sample size with individual differences in clinical characteristics, which limits the generalizability of the research findings. Secondly, the study analyzed specific time‐domain and frequency‐domain indices of HRV, which are commonly analyzed in studies of patients with DOC. Other analytical techniques, including the Poincaré plots [66] and detrended fluctuation analysis [67] or multiscale entropy [68], may offer a more nuanced portrayal of heart rate dynamics and could potentially provide additional insights into predicting changes in consciousness levels among patients undergoing central thalamic DBS.

## Author Contributions

Tianqing Cao: conceptualization, methodology, validation, formal analysis, investigation, writing – original draft, and writing – review and editing. Xiaoke Chai: writing – original draft, writing – review and editing, formal analysis, and visualization. Hongbin Wu: writing – review and editing. Nan wang, Jiuxiang Song: methodology, formal analysis, data curation. Qiheng He: methodology, validation, formal analysis, and writing – review and editing. Sipeng Zhu: formal analysis, visualization. Yitong Jia: methodology, data curation, and visualization. Jizong Zhao: conceptualization, coordinated the research, and finalized the manuscript. Yi Yang: writing – review and editing, project administration, and funding acquisition.

## Conflicts of Interest

The authors declare no conflicts of interest.

## Supporting information


Table S1

Table S2

Table S3

Table S4

Figure S1

## Data Availability

Data are available upon reasonable request. The original data are not yet openly available, as it is being used in ongoing projects. We welcome enquiries about sharing this as part of a collaboration, please contact the corresponding authors.
