# Supplementary material for: Central Thalamic Deep Brain Stimulation Modulates Autonomic Nervous System Responsiveness in Disorders of Consciousness
Source: CNS Neurosci Ther. 2025 Mar 6;31(3):e70274. doi: 10.1111/cns.70274 (PMC11884924; doi:10.1111/cns.70274)
Supplement: Supplementary file 1 — Table S1 [file CNS-31-e70274-s003.docx]

**SUPPLEMENTARY TABLE 1.** One-way repeated measures ANOVA results of time-domain and frequency-domain of HR during DBS-Pre, DBS-On and DBS-Post.

|  | Source of varation | SS | Df | MS | F | *p*-value |
| --- | --- | --- | --- | --- | --- | --- |
| **mRRI** | Between Group | 368039.167 | 2 | 92908.157 | 3.92 | **0.032** |
|  | Within Group | 35508.722 | 33 | 17709.361 |  |  |
| **SDNN** | Between Group | 31036.056 | 2 | 15518.028 | 5.005 | **0.013** |
|  | Within Group | 102322.25 | 33 | 3100.674 |  |  |
| HF | Between Group | 24333.327 | 2 | 16166.583 | 1.859 | 0.113 |
|  | Within Group | 341721.583 | 33 | 10991.563 |  |  |
| **LF** | Between Group | 60256.889 | 2 | 30128.444 | 4.08 | **0.027** |
|  | Within Group | 140429.667 | 33 | 4255.444 |  |  |
| LF/HF | Between Group | 6.587 | 2 | 3.245 | 1.98 | 0.154 |
|  | Within Group | 50.214 | 33 | 11.515 |  |  |
| **TP** | Between Group | 894126.222 | 2 | 387063.111 | 3.079 | **0.041** |
|  | Within Group | 757879.245 | 33 | 229641.788 |  |  |
| nHF | Between Group | 176.222 | 2 | 88.111 | 1.286 | 0.213 |
|  | Within Group | 10166.75 | 33 | 308.083 |  |  |
| nLF | Between Group | 1343.056 | 2 | 671.528 | 2.916 | 0.068 |
|  | Within Group | 7600.583 | 33 | 230.321 |  |  |

HRV: heart rate variability; mRRI: mean R-R interval; SDNN: standard deviation of normal-to-normal intervals; HF: high frequency; LF: low frequency; nHF: normalized high frequency; nLF: normalized low frequency; LF/HF: low to high-frequency ratio; TP: total power.

One-way repeated measures ANOVA ( two-tailed, unpaired) was used to compare the difference;

Bold in the p column indicates a statistical significance with p<0.05.
