# Supplementary material for: Central Thalamic Deep Brain Stimulation Modulates Autonomic Nervous System Responsiveness in Disorders of Consciousness
Source: CNS Neurosci Ther. 2025 Mar 6;31(3):e70274. doi: 10.1111/cns.70274 (PMC11884924; doi:10.1111/cns.70274)
Supplement: Supplementary file 2 — Table S2 [file CNS-31-e70274-s005.docx]

**SUPPLEMENTARY TABLE 2.** Multiple comparison of the mRRI, SDNN, LF and TP during DBS-Pre, DBS-On and DBS-Post.

| HRV Features | Different stages | | Difference of average value | Standard error | *p*-value | 95% CI | |
| --- | --- | --- | --- | --- | --- | --- | --- |
|  |  |  |  |  |  | Lower limit | upper limit |
| mRRI | DBS-On | DBS-Pre | -160.416 | -42.732 | **0.012** | -104.676 | -14.166 |
|  |  | DBS-Post | -144.124 | -28.372 | **0.018** | -98.25 | -17.749 |
| SDNN | DBS-On | DBS-Pre | 55.157 | 26.631 | **0.044** | 15.484 | 39.849 |
|  |  | DBS-Post | 59.247 | 14.631 | **0.011** | 24.817 | 57.182 |
| LF | DBS-On | DBS-Pre | 131.416 | 44.658 | **0.038** | 23.428 | 112.262 |
|  |  | DBS-Post | 87.433 | 61.65 | 0.314 | -13.512 | 82.178 |
| TP | DBS-On | DBS-Pre | 410.636 | 79.636 | **0.041** | 77.358 | 318.692 |
|  |  | DBS-Post | 355.346 | 195.638 | 0.433 | -242.692 | 453.358 |

HRV: heart rate variability; mRRI: mean R-R interval; SDNN: standard deviation of normal-to-normal intervals; HF: high frequency; LF: low frequency; nHF: normalized high frequency; nLF: normalized low frequency; LF/HF: low to high-frequency ratio; TP: total power.

One-way repeated measures ANOVA ( two-tailed, unpaired) was used to compare the difference;

Based on the results of the ANOVA test, post hoc tests using the LSD for multiple comparisons;

Bold in the p column indicates a statistical significance with p<0.05.
