# Supplementary material for: Central Thalamic Deep Brain Stimulation Modulates Autonomic Nervous System Responsiveness in Disorders of Consciousness
Source: CNS Neurosci Ther. 2025 Mar 6;31(3):e70274. doi: 10.1111/cns.70274 (PMC11884924; doi:10.1111/cns.70274)
Supplement: Supplementary file 3 — Table S3 [file CNS-31-e70274-s001.docx]

**SUPPLEMENTARY TABLE 3.** Short-Term Changes in HRV Indices Following Central Thalamic Stimulation (DBS-Pre vs DBS-Post in three days).

|  | HRV Features | U | *p*-value |
| --- | --- | --- | --- |
| Time-domain | mRRI | **20.00** | **0.024** |
|  | SDNN | **21.00** | **0.018** |
| Frequency-domain | HF | **25.00** | **0.025** |
|  | LF | **18.00** | **0.010** |
|  | LF/HF | **28.00** | **0.031** |
|  | TP | 58.00 | 0.419 |
|  | nHF | 53.00 | 0.324 |
|  | nLF | 42.00 | 0.136 |

HRV: heart rate variability; mRRI: mean R-R interval; SDNN: standard deviation of normal-to-normal intervals; HF: high frequency; LF: low frequency; nHF: normalized high frequency; nLF: normalized low frequency; LF/HF: low to high-frequency ratio; TP: total power.

Wilcoxon rank sum test (Mann-Whitney U Test) was used to compare the difference;

Bold in the p column indicates a statistical significance with p<0.05.
