# Supplementary material for: Central Thalamic Deep Brain Stimulation Modulates Autonomic Nervous System Responsiveness in Disorders of Consciousness
Source: CNS Neurosci Ther. 2025 Mar 6;31(3):e70274. doi: 10.1111/cns.70274 (PMC11884924; doi:10.1111/cns.70274)
Supplement: Supplementary file 4 — Table S4 [file CNS-31-e70274-s004.docx]

**SUPPLEMENTARY TABLE 4.** Comparison of changes in HRV indices among patients with effective awakening following six months of DBS follow-up.

| Variable | Outcome | | U |  |
| --- | --- | --- | --- | --- |
|  | Improved (n=3) | Unchanged(n=5) |  | *P*-value |
| ΔmRRI | -30.45±20.03 | -20.14±12.8 | 36.00 | 0.334 |
| ΔSDNN | 10.06±12.63 | 19.23±16.46 | 34.00 | 0.312 |
| ΔHF | -90.38±32.11 | -24±12.97 | **25.00** | **0.024** |
| ΔLF | 172.34±54.59 | 61.84±34.72 | **18.00** | **0.010** |
| ΔLF/HF | 2.73±1.31 | 1.25±0.76 | 51.00 | 0.398 |
| ΔTP | 425.28±122.66 | 322.34±168.57 | **28.00** | **0.041** |
| ΔnLF | 23.08±14.06 | 17.73±11.36 | 53.00 | 0.095 |
| ΔnHF | -14.08±9.06 | -8.73±6.36 | 42.00 | 0.145 |

HRV: heart rate variability; mRRI: mean R-R interval; SDNN: standard deviation of normal-to-normal intervals; HF: high frequency; LF: low frequency; nHF: normalized high frequency; nLF: normalized low frequency; LF/HF: low to high-frequency ratio; TP: total power.

Wilcoxon rank sum test (Mann-Whitney U Test) was used to compare the difference;

Bold indicates a statistical significance with p<0.05.
