# Supplementary material for: Central Thalamic Deep Brain Stimulation Modulates Autonomic Nervous System Responsiveness in Disorders of Consciousness
Source: CNS Neurosci Ther. 2025 Mar 6;31(3):e70274. doi: 10.1111/cns.70274 (PMC11884924; doi:10.1111/cns.70274)
Supplement: Supplementary file 5 — Figure S1 [file CNS-31-e70274-s002.docx]

**SUPPLEMENTARY FIGURE 1.** The SHAP value aims to quantify the marginal contribution of eight features within HRV towards the prediction outcome, specifically illustrating the relative impact of each feature on the predicted output within the current modeling framework. This assessment of contribution is achieved by comprehensively considering the marginal effects generated by the features across all potential combinations of features. SHAP: SHapley Additive exPlanations

**
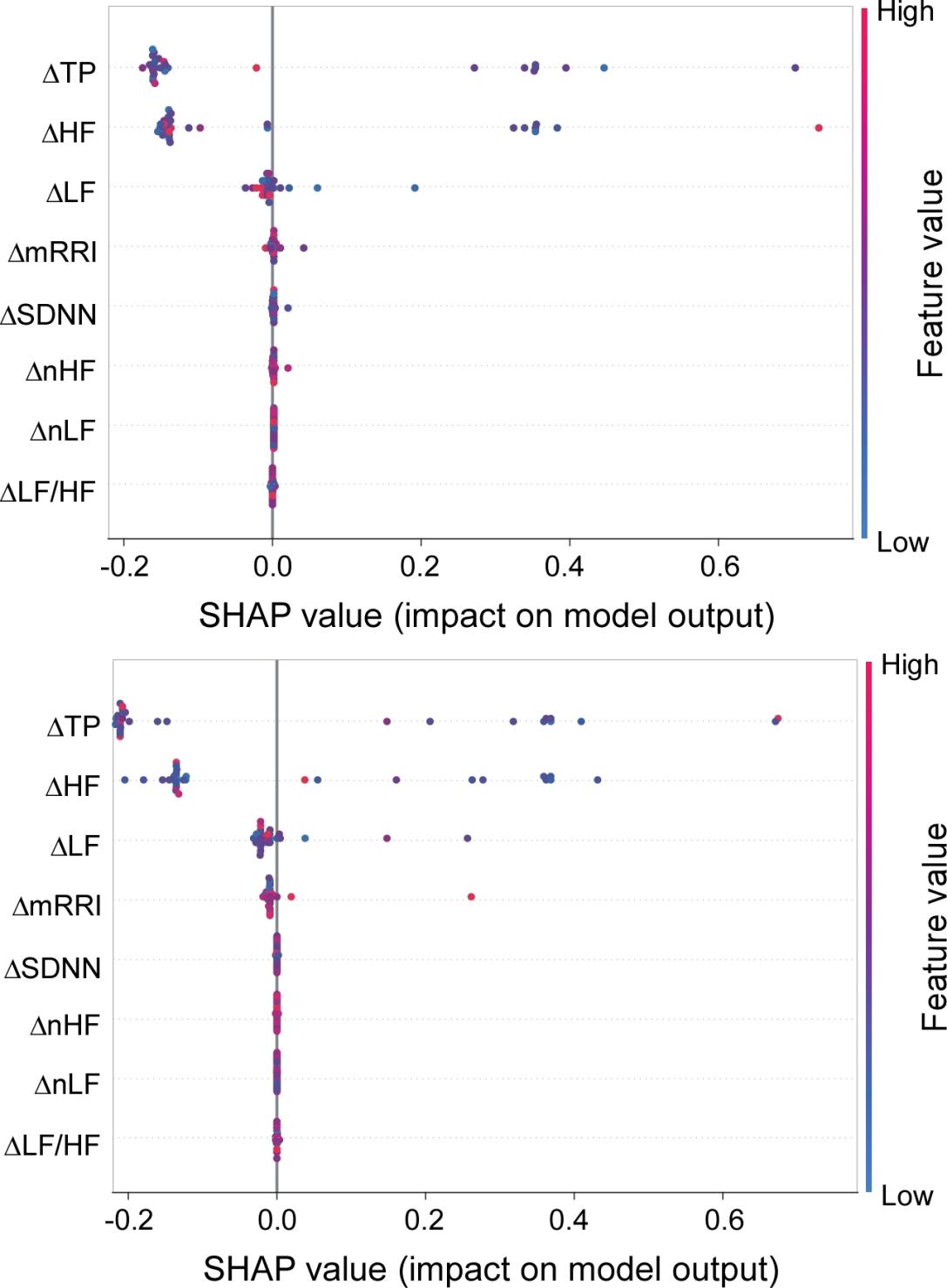

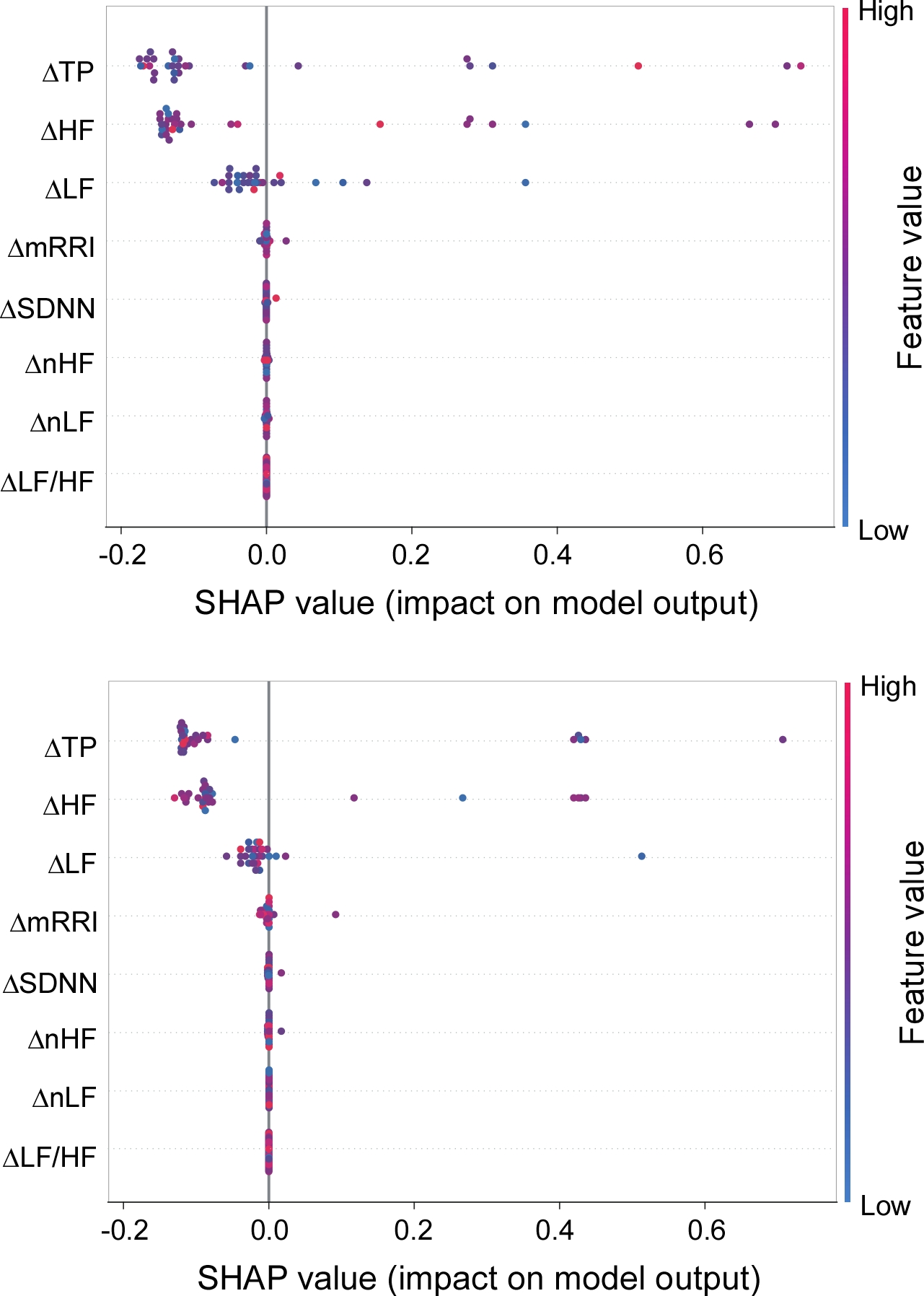

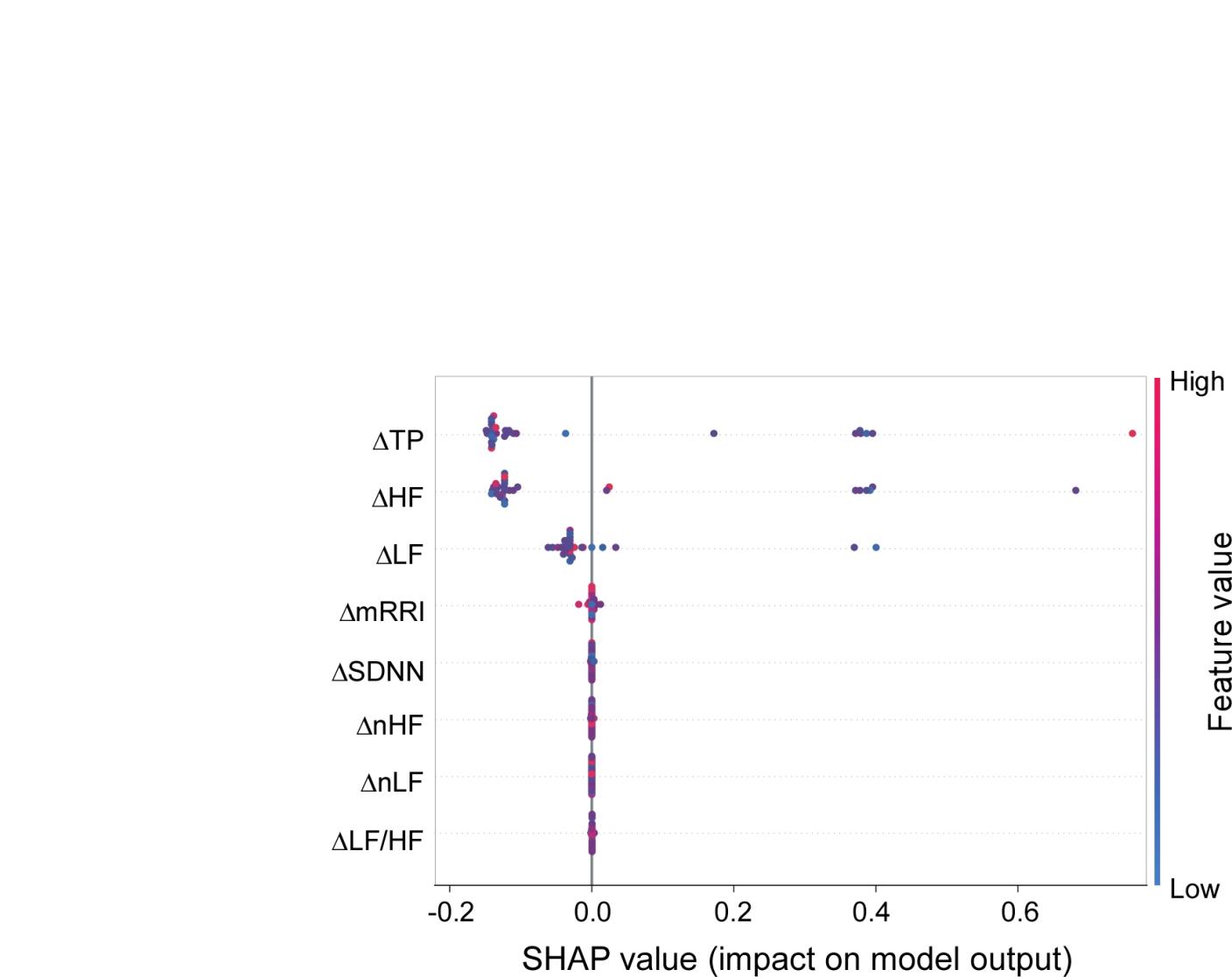
**
